# Advanced multimodal imaging: FLIM, PLIM, and FluoRaman enabled by novel diarylacetylene probes

**DOI:** 10.1039/d5an00953g

**Published:** 2025-12-01

**Authors:** Joshua G. Hughes, Dimitrios Tsikritsis, Alexandra E. Bailie, Camilla Dondi, Eva Dias, Helen H. Fielding, Natalie A. Belsey, Michael Shaw, Stanley W. Botchway, Carrie A. Ambler, John M. Girkin, David R. Chisholm

**Affiliations:** a Department of Chemistry, University College London 20 Gordon Street London WC1H 0AJ UK; b Centre for Advanced Instrumentation, Department of Physics, University of Durham Lower Mountjoy South Road Durham DH1 3LS UK; c LightOx Ltd 65 Westgate Road Newcastle upon Tyne NE1 1SG UK david.chisholm@lightox.co.uk; d Chemical and Biological Sciences Department, National Physical Laboratory Hampton Road Teddington Middlesex TW11 0LW UK; e Central Laser Facility, STFC Rutherford Appleton Laboratory Didcot Oxfordshire OX11 0QX UK; f Hawkes Institute and Department of Computer Science, University College London 66-72 Gower St London WC1E 6EA UK; g Department of Biosciences, University of Durham South Road Durham DH1 3LE UK

## Abstract

In drug design, understanding the subcellular localisation and physicochemical behaviour of candidate molecules is essential for optimising their efficacy and elucidating their mechanisms of action. Imaging probes are routinely employed for this purpose, though most studies rely on a single imaging modality. By integrating multiple imaging techniques into a multimodal system, a more comprehensive understanding of localisation, microenvironment, and physicochemical interactions can be achieved. Yet, there remains a scarcity of specialised imaging probes with well-defined photophysical profiles, especially those that combine luminescence with a Raman-active tag in the cell-silent region, where signal clarity is maximised. Such probes could function independently or be conjugated to drugs or targeting moieties as dual-mode imaging tags. In this study, we showcase the application of a suite of advanced imaging modalities: fluorescence microscopy (CLSM); Fluorescence Lifetime Imaging Microscopy (FLIM); Phosphorescence Lifetime Imaging Microscopy (PLIM); and simultaneous fluorescence and Raman spectroscopy (FluoRaman). The modalities were exemplified by a series of novel, highly solvatochromic diarylacetylene-based photosensitisers that feature alkyne Raman tags and exhibit diverse subcellular localisation.

## Abstract 2

Integrated fluorescence, lifetime, and Raman imaging of solvatochromic diarylacetylene probes provides complementary insight into subcellular localisation, microenvironment and physicochemical interactions.

## Graphical abstract

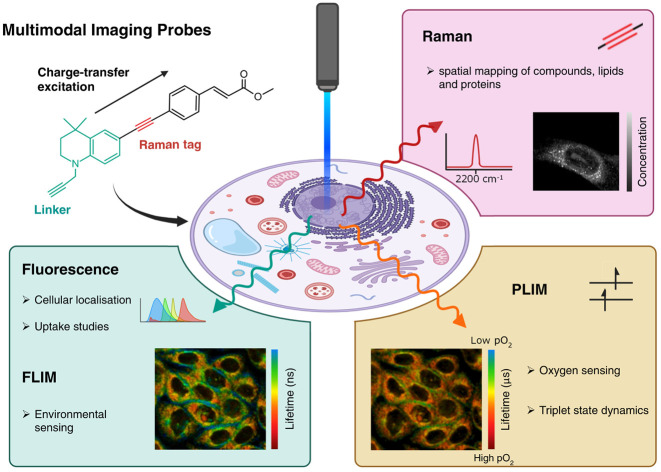

## 1. Introduction

Microscopy techniques are routinely employed in drug discovery to investigate uptake, spatial distribution, molecular interactions, and mechanisms of action. To achieve therapeutic efficacy, drug molecules must possess physicochemical properties that enable efficient tissue and/or cellular uptake and targeted localisation to specific subcellular regions. To monitor these processes, drugs are often tagged with small molecular labels – such as fluorophores, radioisotopes, or Raman-active tags – to provide critical information about their behaviour in biological systems. However, most of these strategies rely on a single imaging modality, which limits the depth of information that can be obtained and often fails to capture the full complexity of the system.

Fluorescence intensity-based microscopy techniques are a common method of experimentation due to their spatiotemporal resolution combined with a low limit of detection. Fluorescence Lifetime Imaging Microscopy (FLIM) uses time-gated methods to record the arrival time of an emitted photon to generate image contrast. As fluorescence lifetime is largely independent of probe concentration – notwithstanding self-quenching and reabsorption effects at high concentrations – it provides an absolute, reproducible metric across varying experimental set-ups. Moreover, the fluorescence lifetime of the probes can be highly sensitive to the local microenvironment, with lifetimes varying in response to changes in factors such as polarity,^1^ pH,^2^ temperature, and viscosity.^3^ This sensitivity makes FLIM a valuable tool for environmental sensing in biological systems when used with well-characterised environmentally-sensitive probes.^4^ Due to these advantages, FLIM has found wide application in research and clinical settings, including metabolic imaging *via* autofluorescence of NADH and FAD,^5,6^ diagnostics and disease monitoring,^7–9^ fluorescence-guided surgery,^10^ and FLIM-FRET studies of protein–protein interactions.^11^

While FLIM typically measures fluorescence lifetimes on the picosecond-to-tens-of-nanoseconds timescale, Phosphorescence Lifetime Imaging Microscopy (PLIM) operates on the hundreds-of-nanoseconds-to-tens-of-microseconds range. Phosphorescence arises from excited triplet states formed *via* intersystem crossing (ISC) from the excited singlet state. As the return to the singlet ground state is spin-forbidden, this radiative decay pathway is slower than fluorescence. Using beam blanking, FLIM and PLIM measurements can be acquired simultaneously from the same sample. Although fluorescent states are oxygen-sensitive, phosphorescent states in many probes are particularly susceptible to quenching, resulting in shorter lifetimes and weaker phosphorescence. This dual imaging modality enables the simultaneous study of different environmental factors, such as cellular metabolism (*via* FLIM of NAD(P)H) and oxygen tension (*via* PLIM of extrinsic dyes).^13–17^ These approaches are particularly relevant in cancer research, where hypoxic conditions are common.^18^ The most common PLIM probes incorporate transition-metal complexes^19–21^ to enhance spin–orbit coupling *via* the heavy-atom effect.^22^ Increased spin–orbit coupling facilitates intersystem crossing (ISC) by enabling interaction between spin and orbital angular momentum, thereby allowing transitions between states of differing spin multiplicities. One such probe, an iridium(iii) complex developed by Zhao, Lo, Huang, and co-workers can distinguish between hypoxic, normoxic, and hyperoxic environments through dual phosphorescent emission.^23^ Furthermore, photosensitisers used in photodynamic therapy (PDT) are designed to maximise high triplet quantum yields and reactive oxygen species (ROS) from their triplet states. Thus, both oxygen consumption and metabolic activity can be monitored in tandem.^24^

Mapping the spatial distribution of probe concentrations in biological tissues remains a major challenge. While fluorescence lifetime is largely concentration-independent at low concentrations,^25^ fluorescence intensity is not and can be influenced by environmental factors – especially in probes with charge-transfer character. In contrast, stimulated Raman spectroscopy (SRS) provides a signal proportional to concentration and is less affected by the microenvironment. Thus, it offers a relative quantitative readout of local probe levels. FluoRaman imaging, which combines Raman spectroscopy and fluorescence, enables multimodal, three-dimensional chemical mapping alongside real-time probe tracking. This technique has promising applications in pharmacokinetics, toxicology, and histology.^26–28^ Since Raman signals occur on the picosecond timescale, they can be temporally gated from photoluminescence, allowing separation of the two modalities. Raman imaging is label-free and non-destructive, capable of detecting lipids, proteins, and nucleic acids. Probes with strong Raman signals in the so-called ‘cell-silent’ region (1800–2600 cm^−1^) can be imaged with minimal background interference. Raman-active tags such as alkyne (C≡C) and nitrile (C≡N) groups have been used to track small molecules in live cells.^29,30^ However, multifunctional probes that combine strong fluorescence with strong Raman activity are still rare. Only a few have been reported – for example, Li *et al.* developed a mitochondrial probe bearing both alkyne and nitrile tags,^31^ and Lin *et al.* designed a nitrile-tagged lipid droplet probe.^32^

By integrating multiple imaging modalities, the strengths of each technique can complement and enhance each other while mitigating their individual limitations. This multimodal approach enables a more comprehensive and nuanced understanding of molecular behaviour in biological systems. Fluorescence Lifetime Imaging Microscopy (FLIM) provides detailed information about the local microenvironment, including polarity and viscosity. Phosphorescence Lifetime Imaging Microscopy (PLIM) offers a powerful means to probe triplet states, using well-characterised phosphorescent probes to infer relative oxygen concentrations and detect hypoxic or oxidative stress conditions. Raman microscopy, meanwhile, delivers precise spatial mapping of probe or drug distribution – critical for assessing cellular uptake and localisation at both the subcellular and tissue level. Such techniques rely on well-characterised probes, as incomplete or inaccurate photophysical data can compromise interpretation. Achieving this level of characterisation demands a broad suite of measurements, since even small changes in chemical structure – especially in small molecules – can profoundly alter photophysical behaviour and physicochemical properties.^33^

**Fig. 1:**
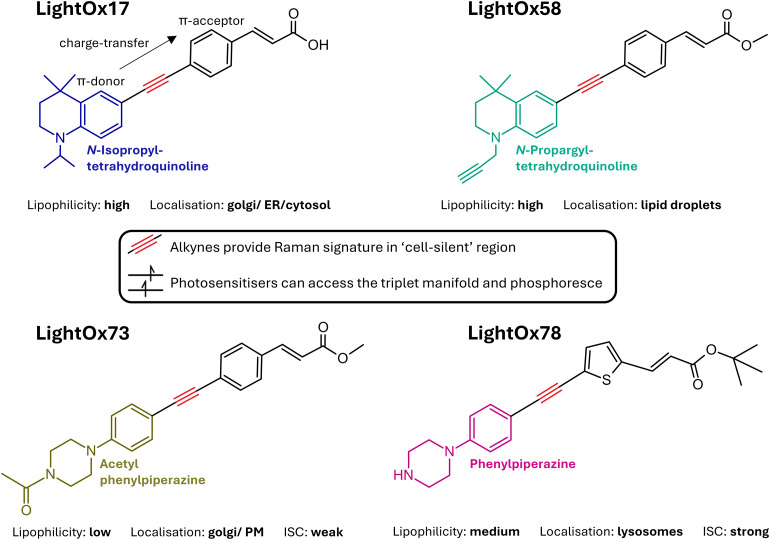
Chemical structures of LightOx compounds.^12^

Diarylacetylene-based photosensitisers (Fig. 1) are uniquely suited for advanced microscopy techniques such as FLIM, PLIM, and Raman imaging. These π-donor–acceptor molecules undergo charge-transfer upon excitation, resulting in photophysical properties that are inherently sensitive to their microenvironment. Coupled with diverse and often broad subcellular localisation, they are promising candidates for environmental sensing using FLIM. Their ability to populate the triplet state also makes them compatible with PLIM, where long-lived triplet emission enables oxygen-sensitive imaging. Additionally, the central acetylene moiety exhibits a characteristic vibrational stretch within the ‘cell-silent’ region, making these compounds suitable for Raman and FluoRaman microscopy. A broad library of such diarylacetylene derivatives – featuring varied subcellular localisation patterns, physicochemical properties, and photophysical behaviours – has been previously reported.^27,34–36^ Here, we exemplify the application of the complementary imaging techniques: FLIM; PLIM; and FluoRaman, using four representative molecules drawn from this library, chosen for their differing lipophilicities, localisation profiles, and intersystem crossing efficiencies.

## 2. Methods

### 2.1. Synthesis

Solvents and reagents used in the experiments were purchased from Sigma-Aldrich and Life Technologies and were used without any further purification. Compounds were prepared using existing methods.^27,36,37^

### 2.2. Cell lines and culture

Squamous cell carcinoma 4 (SCC-4) purchased from ATCC (Cat. No. CRL-1624). They were cultured at 37 °C/5% CO_2_ and 95% relative humidity in DMEM:F12 medium (Gibco Cat. No. 11330-032) containing 10% foetal bovine serum (ThermoFisher, Cat. No. 10270-106), 400 ng ml^−1^ hydrocortisone (Merck Cat. No. H0888-1G) and 1% penicillin/streptomycin (ThermoFisher, Cat. No. 15070-063).

Chinese hamster ovary (CHO) cells were obtained from the European Collection of Cell Cultures. CHO cells were cultured in DMEM medium (Gibco) containing 10% foetal calf serum, penicillin (100 units per mL), streptomycin (100 μg mL^−1^) and glutamine (2 mM). For microscopy, adherent cell cultures were grown in glass-bottom 8-well plates and incubated at 37 °C in a humidified atmosphere containing 5% CO_2_.

### 2.3. Cell lines and staining

For confocal laser scanning microscopy (CLSM) and FluoRaman experiments, SCC-4 cells were incubated with 50 μM compound concentrations (0.5% v/v DMSO, diluted from a 10 mM stock) for 15 minutes at 37 °C/5% CO_2_. Cells were then fixed with 4% paraformaldehyde (pre-warmed to 37 °C) for 10 minutes at room temperature.

For two-photon FLIM, CHO cells were incubated with 1 μM compound concentration (0.1% v/v DMSO, diluted from a 1 mM stock) for 15 minutes at 37 °C/5% CO_2_ and imaged live in PBS. For PLIM, cells were incubated with 5 μM (0.5% v/v DMSO, diluted from a 1 mM stock) for 30 minutes.

### 2.4. Multi-photon microscopy

The microscopy set-up at Central Laser Facility was custom built. The lasers for single and multi-photon excitation were a Ti:Sapphire oscillator, Mai Tai HP DeepSee (Spectra Physics), tuning range 690–1040 nm. The laser has an average laser power of 2.4 W at 800 nm and pulse length of ∼100 fs. The laser repetition rate is 80 MHz ± 1 MHz. The laser was focused using a 60× Nikon water immersion objective with a numerical aperture of 1.27. Fluorescence was collected by the same objective and filtered using a BG39 and 520 ± 30 nm band pass filters. Spectra were measured with a Acton 275 Spectrometer and fluorescence lifetimes were measured with a Becker and Hickl SPC-830 TCSPC system. This system was used for two-photon cross section calculations, fluorescence lifetime calculations and two-photon FLIM. The laser was tuned for 780 nm two-photon FLIM.

### 2.5. Phosphorescence lifetime imaging microscopy (PLIM)

The Phosphorescence Lifetime Imaging Microscopy (PLIM) used in this study consisted of a DCS120 confocal laser scanning unit coupled to a Nikon Ti-E inverted microscope together with a time correlated single-photon counting PCI card (SPC150, Becker & Hickl GmBH). We used a ‘beam blanking multiplex mode’ with a high repetition rate diode laser (80 MHz). In this configuration, fluorescence was collected over the first 10 ns of the decay, before the laser is effectively switched off, so the remaining pixel dwell on the μs range can collect the phosphorescence. The scanning process and phosphorescence decay acquisition was controlled by the TCSPC software SPCM v 9.77. Samples were excited with 405 nm or 488 nm, with 40 ps pulse length. PLIM Images were acquired with a 60× (NA 1.20) water immersion objective. Phosphorescence emission was acquired using a Becker & Hickl HPM100-40 hybrid detector. Two 435 nm long-pass filters (provided by Becker & Hickl GmBH) were used to eliminate the excitation wavelength. PLIM images were acquired at 256 × 256 pixels with each pixel containing a full fluorescence and phosphoresce decay profile. The data were analysed using Becker & Hickl SPCImage V.8.8.

### 2.6. Fluorescence lifetime

Fluorescence lifetimes of the molecules were measured outside of a cellular environment in CHCl_3_, EtOH and toluene for 10 μM and 50 μM compound concentrations.

Glycerol and sucrose were used to vary the viscosity of the solutions. 0, 20, 40% v/v glycerol solutions in CHCl_3_ were made with 50 μM LightOx17 and measured using two-photon excitation at 780 nm. 0, 20, 40, 60% v/v sucrose solutions in water were made with 30 μM LightOx17 and measured using one-photon excitation at 405 nm. Samples were visually analysed for homogeneity.

Fluorescence lifetime data were analysed for single and bi-exponential decays using Becker & Hickl SPMC and FLIMfit^38^ software. The residuals and associated *χ*^2^ values were considered when selecting the optimal fit.

### 2.7. Indicative two-photon cross sections

Two-photon cross sections *σ*_2,S_, for sample, S, and reference, R, were measured using a comparative method against a known reference, as shown in equation (eqn (1)).^39^1
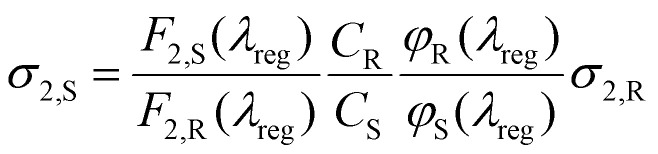


Here, *C* is the concentration, *F*_2_ is the fluorescence under two-photon excitation (TPE) at the registration wavelength, *λ*_reg_, and *φ* is the differential quantum efficiency defined as:2
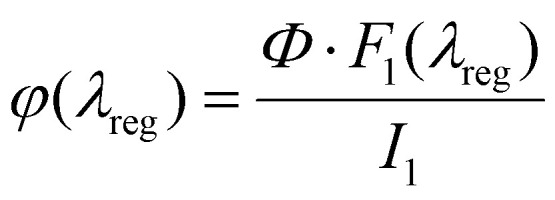
where *Φ* is the quantum yield, *F*_1_ is the fluorescence intensity under one-photon excitation (OPE) at the registration wavelength *λ*_reg_, and *I*_1_ is the integrated fluorescence across the entire emission spectrum from OPE. OPE measurements were performed at 405 nm, while TPE measurements were conducted across 720–880 nm.

For measurements in chloroform and toluene, LightOx17 in toluene was used as the reference,^35^ whereas for measurements in ethanol, Rhodamine B in ethanol was used.^39^

### 2.8. Spontaneous Raman

Spontaneous Raman spectra were recorded with a Renishaw InVia Qontor spectrometer. A 100 ms acquisition was used with a 50 × 0.5 NA objective. Two excitation wavelengths were used; 532 nm and 830 nm. For 532 nm excitation, the laser power was set to 13.7 mW, 100 accumulations per acquisition and a grating of 2400 lines per mm. For 830 nm excitation, the laser power was set to 7.0 mW, 200 accumulations per acquisition and a grating of 1200 lines per mm. 3 repeats were taken per sample. The acquisition software was Renishaw WiRE version 5.4. Cosmic rays were removed prior to baseline subtraction (intelligent polynomial order 12, noise tolerance 1.50) and spectra normalised to the highest intensity peak.

### 2.9. Confocal microscopy

Confocal laser scanning microscopy was performed using a Leica Stellaris 5 system controlled using LASX software (version 4.7.0.28176). Images were acquired under 405 nm excitation, with an emission bandpass of 480–550 nm, using a 63×/1.40 oil immersion objective lens (HC PL APO CS2). Transmitted light (differential interference contrast, DIC) images were also acquired to allow visualisation of cell bodies and lipid droplets (LDs) without additional staining. To facilitate visualisation, focal series (*z*-stacks) were processed into 2D maximum intensity projections using Fiji (ImageJ).

### 2.10. FluoRaman

FluoRaman (SRS, CARS and two-photon fluorescence) images were acquired on a Leica SP8 laser scanning microscope coupled to a PicoEmerald-S laser system.^40^ The PicoEmerald-S outputs two pulsed 2 ps laser beams: a 1031.2 nm Stokes beam which was spatially and temporally overlapped with a tunable pump beam. The Stokes beam was modulated at 20 MHz and stimulated Raman loss signals were detected in transmission using a lock-in amplifier (UHFLI, Zurich instruments). Signals from TPEF were *epi*-detected complementary in a separate PMT channel, simultaneously to SRS. Images were acquired with a water immersion 40× magnification lens (1.1NA, Leica) used in conjunction with an air condenser lens (0.9NA, Leica). The laser power was set to 45% which corresponds to approximately 52.9 mW for the pump beam and 30 mW for the Stokes beam at the sample. The acquisition software was Leica LAS-X version: 3.5.7.23225, and subsequent analysis was performed in ImageJ.

The pump laser was tuned to various vibrational modes: 2210 cm^−1^ for LightOx58 and LightOx78, 2850 cm^−1^ for the CH_2_ symmetric stretching of lipid molecules, 2945 cm^−1^ for the CH_3_ asymmetric stretching of lipids and proteins,^41^ and finally 2650 cm^−1^ for the off-resonance control.

For the confocal and FluoRaman microscopy, SCC-4 cells were plated on gridded dishes (Mat Tek, Cat. No. P50G-1.5-14-FGRD) and treated with 50 μM compound concentrations. Cells were fixed with 4% PFA for 10 minutes prior to imaging.

## 3. Results

### 3.1. Photophysical characterisation

The four compounds studied are diarylacetylenes that undergo a charge-transfer (CT) process between π-donor and π-acceptor groups upon excitation. As expected for CT systems, they exhibit characteristic photophysical properties (Table 1), including highly solvatochromic fluorescence emission and solvent polarity-dependent quantum yields and fluorescence lifetimes.^27,34,35^ In moderately polar solvents such as toluene and chloroform, blue-shifted emission is observed along with higher quantum yields (*e.g.*, 0.58 for LightOx78 in toluene) and longer fluorescence lifetimes (1.64 ns for LightOx78 in toluene). In contrast, in more polar solvents such as ethanol, red-shifted emission is accompanied by very low quantum yields (0.015 for LightOx78) and short lifetimes (144 ps for LightOx78). These effects arise from a combination of solvent-induced aggregation and solvent relaxation mechanisms. Aggregation can lead to self-quenching and non-radiative decay, while solvent relaxation stabilizes the S_1_ excited state through reorientation of solvent molecules around the excited-state dipole, reducing the energy gap between the S_1_ and S_0_ states. According to the energy gap law,^42^ this can cause enhanced non-radiative decay and shorter lifetimes.

**Table 1:** Photophysical properties of LightOx17, LightOx58, LightOx73 and LightOx78 in toluene, chloroform and ethanol

Compound	Solvent	*λ*_abs_ (max)/nm	*ε*/M^−1^ cm^−1^	*λ*_em_ (max)/nm	*Φ*	〈*τ*〉/ns
LightOx17	Toluene	390	27 032	500	0.67	2.00
	Chloroform	398	23 924	572	0.76	1.62
	Ethanol	387	29 616	525	0.055	0.443
LightOx58	Toluene	377	32 110	465	0.82	1.62
	Chloroform	379	34 133	527	0.46	2.40
	Ethanol	375	44 440	548	0.006	0.099
LightOx73	Toluene	361	38 363	468	0.94	1.40
	Chloroform	359	32 826	504	0.72	2.12
	Ethanol	357	39 155	535	0.008	0.105
LightOx78	Toluene	387	36 688	486	0.58	1.64
	Chloroform	383	24 086	527	0.50	2.33
	Ethanol	385	46 282	562	0.015	0.144

The wide range of fluorescence lifetimes observed underscores the environmental sensitivity of this parameter.^42^ The polarity dependence of lifetime is particularly significant given that these compounds display diverse subcellular localisation (*vide infra*) in biological systems to regions with varying lipophilicities. As lipophilicty correlates with polarity,^43^ they present promising candidates for environmental sensing *via* fluorescence lifetime imaging. In addition to polarity, fluorescence lifetime is also influenced by other factors such as pH, oxygen concentration, temperature, and viscosity. The impact of viscosity was further explored (SI Fig. 1). Typically, increased viscosity leads to longer fluorescence lifetimes due to restricted molecular rotation.^44^ This was hypothesised to apply to the LightOx series, as the efficiency of fluorescence (lifetime and quantum yield) could be affected by the rotational freedom of the central acetylene moieties.

To test this, glycerol was added to chloroform to raise the solution's viscosity (SI Fig. 1). Contrary to expectations, shorter fluorescence lifetimes were observed with increasing glycerol concentration. This could be due to hydrogen bonding of the carboxylic acid, and subsequent quenching, as observed in fluorescein,^45^ or the high polarity of glycerol, which may promote aggregation and non-radiative decay. Conversely, when sucrose (a larger molecule with lower polarity) was used to increase the viscosity of aqueous solutions, fluorescence lifetimes increased with sucrose concentration (SI Fig. 1).

### 3.2. Indicative two-photon cross sections & Raman spectroscopy

To assess the suitability of the LightOx compounds for advanced imaging modalities – specifically two-photon fluorescence lifetime microscopy and FluoRaman imaging – indicative two-photon absorption cross sections (*σ*_2,S_) and Raman spectra were measured. The *σ*_2,S_ values generally followed the trends observed in fluorescence quantum yields (QYs), decreasing with increasing solvent polarity. The LightOx series exhibited particularly strong two-photon absorption in toluene and chloroform, with values likely exceeding those of standard dyes such as fluorescein (36 ± 5 GM at 800 nm in H_2_O) and Rhodamine B (95 ± 13 GM at 780 nm in methanol).^39^ In contrast, the cross sections were significantly lower in ethanol throughout the LightOx series. This reduction is consistent with the one-photon quantum yields, which are factored into the two-photon cross section calculation (eqn (1)). Overall, the LightOx compounds demonstrate strong two-photon absorption in non-polar solvents, highlighting their promise as two-photon microscopy probes given their affinity for lipophilic environments.

**Fig. 2:**
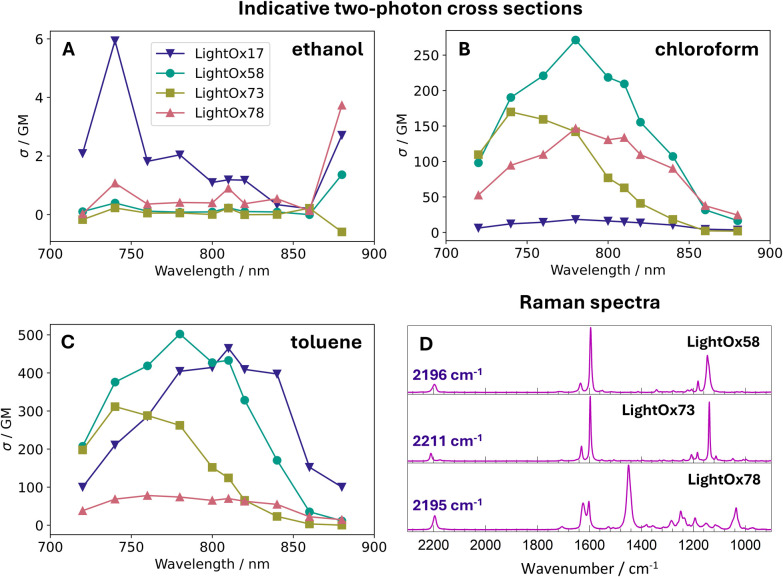
Indicative two-photon cross section of LightOx compounds in (A) ethanol, (B) chloroform and (C) toluene. (D) Raman spectra of LightOx compounds in the solid state with the peak wavenumber of the alkyne (C≡C) stretch labelled.

Raman spectra of the LightOx compounds were acquired in the solid state (Fig. 2D). All compounds exhibited a prominent peak around 2200 cm^−1^, corresponding to the alkyne (C≡C) stretch. This signal lies within the ‘cell-silent’ region of the Raman spectrum (1800–2600 cm^−1^), where endogenous biological signals are minimal. Therefore, any Raman signal observed in this region can be more confidently attributed to the presence of the LightOx molecule, making these compounds suitable for Raman and FluoRaman imaging applications.

### 3.3. Fluorescence lifetime imaging microscopy (FLIM)

**Fig. 3:**
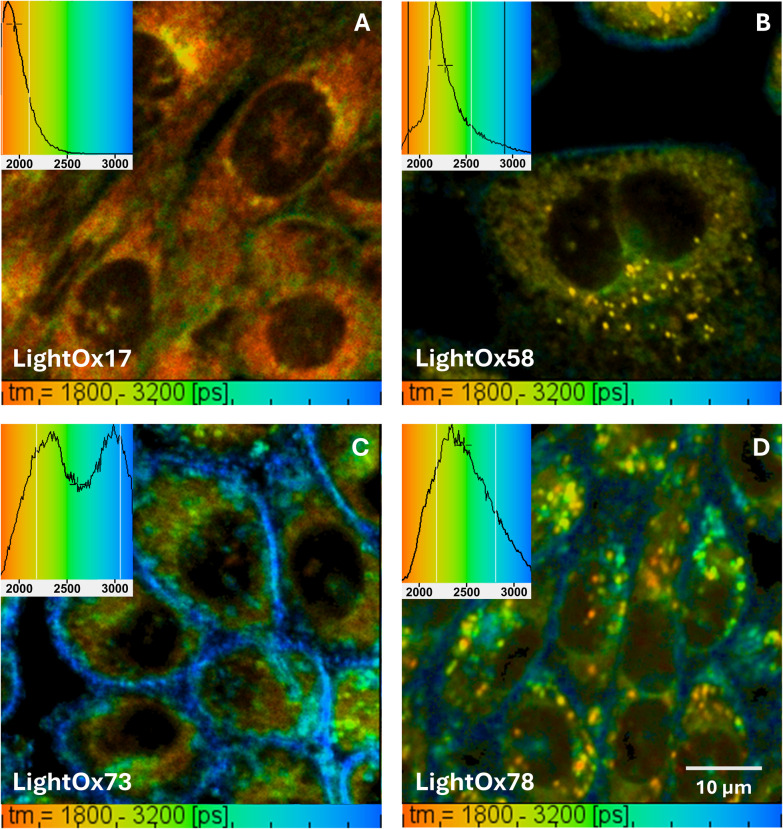
Two-photon FLIM of 1 μM (A) LightOx17, (B) LightOx58, (C) LightOx73 and (D) LightOx78 in live CHO cells. Corresponding lifetime histograms are superimposed onto each image. Lifetime colour maps were set to 1800–3200 ps for all images to aid comparability. The excitation wavelength was 780 nm.

After the two-photon cross sections and fluorescence lifetimes were characterised in solvent systems, two-photon FLIM was conducted for the LightOx compounds in live CHO cells. Fig. 3 displays the compounds with the colourbar set between 1800–3200 ps for comparability. Optimal lifetime ranges for each compound can be found in the SI (Fig. 2). All compounds showed broad cytoplasmic localisation with a wide range of corresponding lifetimes, indicating a high degree of environmental sensitivity, suggesting that the compounds are localised in a diverse range of intracellular environments. Significantly longer lifetimes were associated with the plasma membrane and cellular periphery compared to the cytoplasm and other subcellular regions.

The more lipophilic LightOx17 and LightOx58 (which have tetrahydroquinoline donor moieties) exhibited shorter lifetimes compared to LightOx73 and LightOx78 (phenylpiperazine donors). LightOx17 exhibited the shortest lifetimes overall, with longer values in the perinuclear region (likely ER and Golgi) compared to the cytosol. LightOx73 displayed two discrete lifetime populations of similar intensity, with longer lifetimes in the plasma membrane relative to the cytosol. LightOx58 and LightOx78 have been shown to localise to lipid droplets (LD) and lysosomes, respectively.^46^ The lifetimes of the LDs in LightOx58 had little variation, suggesting a largely uniform environment among these structures. Conversely, the lifetimes of the punctate structures (lysosomes) in LightOx78 showed significant variation, indicating greater heterogeneity.

**Fig. 4:**
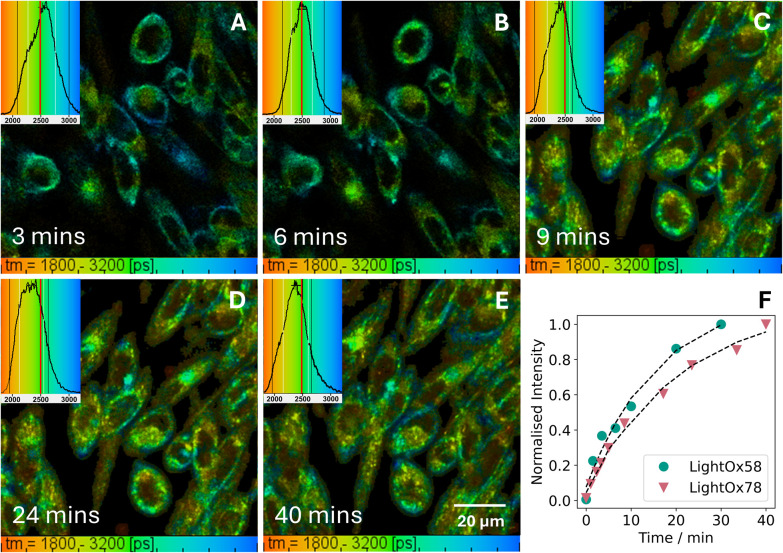
Two-photon FLIM during uptake of (A–E) 1 μM LightOx78 at various time points in live CHO cells. Corresponding normalised intensity plot against time of (F) LightOx78 and LightOx58. The excitation wavelength was 780 nm.

To understand compound uptake, a time series was performed for LightOx58 and LightOx78 (Fig. 4). Lifetimes were longer at earlier time points (<9 minutes), correlating with compound uptake and passage through the plasma membrane. At later time points (>9 minutes), the change in lifetime to shorter timescales is indicative of the cellular localisation of the compounds, with a significant population now within the lysosomes (Fig. 3D).

### 3.4. Phosphorescence lifetime imaging microscopy (PLIM)

This class of novel diarylacetylenes function as photosensitisers.^27,34–37,47^ Following excitation to the S_1_ state, they can undergo intersystem crossing (ISC) to the triplet manifold.^46^ From this triplet state, they are capable of producing reactive oxygen species *via* electron and energy transfer, thereby eliciting a therapeutic response characteristic of photodynamic therapy. However, triplets can also undergo a radiative decay mechanism in the form of phosphorescence.

**Fig. 5:**
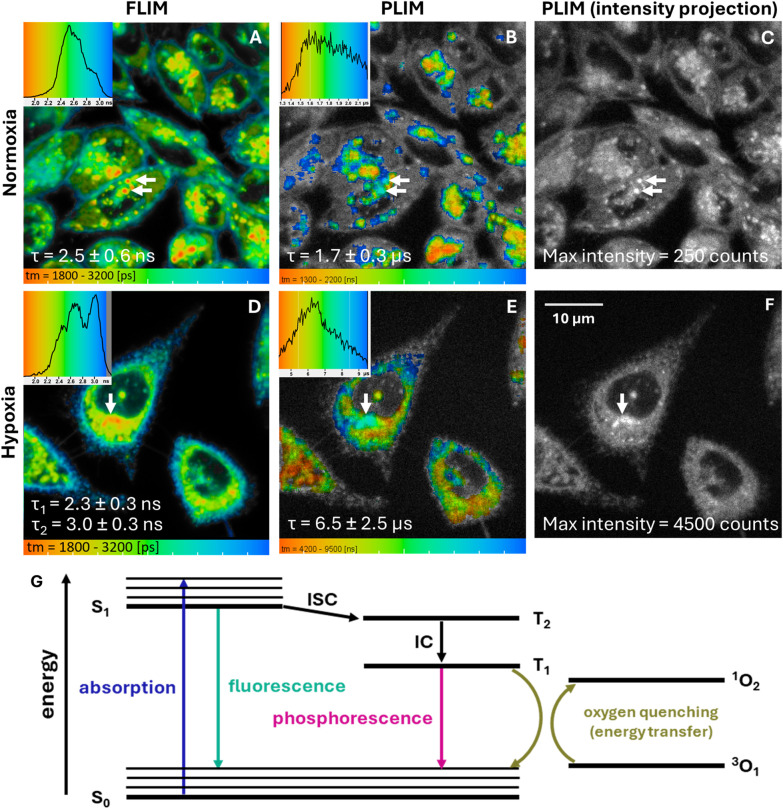
(A and D) FLIM, (B and E) PLIM and (C and F) PLIM maximum intensity projections of 5 μM LightOx78 in live CHO cells. Corresponding lifetime histograms are superimposed onto each image. The excitation wavelength was 405 nm. Note that the FLIM measurements are on the nanosecond timescale, whereas the PLIM measurements are on the microsecond timescale. (G) Jablonksi diagram depicting the fluorescence, phosphorescence and oxygen quenching mechanism.

To investigate the role of oxygen, LightOx73 (a poor photosensitiser) and LightOx78 (a strong photosensitiser)^46^ were imaged under both normoxic and hypoxic conditions (Fig. 5). As photosensitisers, the triplet state of LightOx compounds is effectively quenched by oxygen. Quenching results in a reduced phosphorescent emission and a shorter lifetime.^48^ As expected, LightOx73 (not shown) did not produce detectable phosphorescence under normoxia or hypoxia, consistent with its poor ISC and subsequent photosensitisation efficiency and concomitant low photosensitisation potency.^46^ In contrast, LightOx78, which exhibits efficient ISC, produced a weak phosphorescence emission from lysosomes under normoxia, which was greatly amplified under hypoxia, extending to the cytoplasm.

The impact of oxygen on the phosphorescence lifetime was stark: increasing from 1.7 in normoxic to 6.5 μs in hypoxic conditions. Under normoxic conditions, phosphorescence was detectable only in regions corresponding to the lysosomes, where both fluorescence intensity and compound concentration were highest. In contrast, under hypoxic conditions, phosphorescence was measured throughout the cytosol, although it did not extend to the plasma membrane, where the fluorescence signal was lower.^46^

Determining whether the phosphorescence signal originates from monomeric or aggregated species in cellular environments is not straightforward. Although the staining concentration is below the kinetic solubility of the compounds, once inside cells heterogeneous local concentrations can arise in different organelles, potentially leading to aggregation. Aggregation can facilitate intersystem crossing (ISC) through aggregate-induced ISC mechanisms.^49^ However, the observed phosphorescence intensity correlates linearly with the fluorescence signal (Fig. 5 and SI Fig. 3), which is more consistent with the emission of monomeric species. In addition, N_2_-purging led to increased phosphorescence intensity and prolonged lifetimes, indicative of oxygen-quenched monomeric triplet states. In contrast, aggregates would typically be expected to display reduced oxygen sensitivity as a result of restricted oxygen diffusion into the aggregate structure.

### 3.5. Confocal laser scanning microscopy (CLSM)

**Fig. 6:**
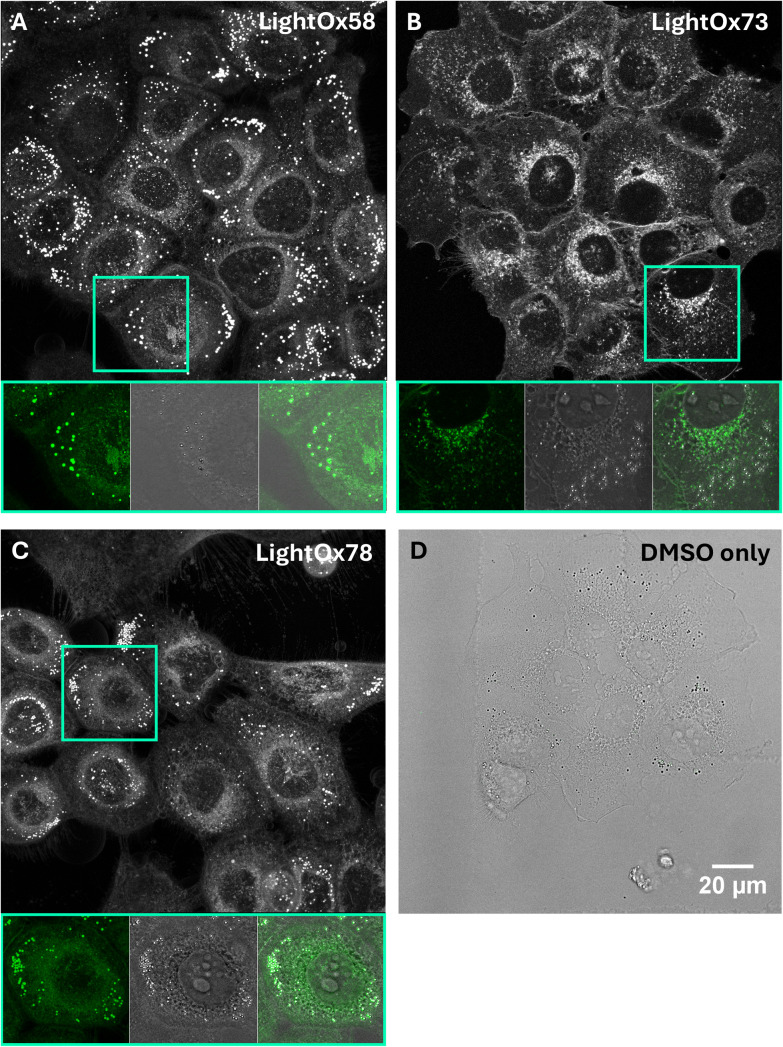
CLSM images of 50 μM (A) LightOx58, (B) LightOx73, (C) LightOx78, and (D) DMSO only (transmitted image) in fixed (4% PFA) SCC-4 cells. Images are max intensity projections from *Z* stacks. The boxed panels show the fluorescence (green), transmitted light (DIC) image (grey scale), and the corresponding overlap.

Before conducting stimulated Raman scattering (SRS) on LightOx compounds in fixed SCC-4s, their localisation was first evaluated *via* confocal microscopy to confirm patterns observed in live cells. Previous work^36^ investigated these compounds in live SCC-4s at lower concentrations, comparing their localisation by co-staining with known subcellular probes. Raman spectroscopy requires higher compound concentrations because it is a less efficient process than fluorescence, relying on transitions through virtual energy states.^50^ Given that some compounds exhibit differential fluorescence localisation between live and fixed cells, it was prudent to image these compounds in fixed cells using confocal microscopy (Fig. 6). Although the compounds were not incubated with co-stains for quantitative comparison, qualitative inferences regarding their fixed-cell localisation could be drawn.

LightOx58 exhibited a very similar fluorescence profile in both live and fixed cells (SI Fig. 4), characterised by intense lipid droplet (LD) staining and dimmer *peri*-nuclear fluorescence. The intense spots in the fluorescence channel correlated with small, round structures in the transmitted channel, indicating that these were also likely LDs. LDs have a higher refractive index than the surrounding cell, and are thus visible in transmitted light (DIC) images.^51^

LightOx73 had strong fluorescence in the cell periphery, plasma membrane, and *peri*-nuclear region in live cells.^36^ In fixed cells, the plasma membrane intensity remained, with intense punctate fluorescence in the perinuclear regions. Again, the vast majority of the fluorescent spots did not correlate with the structures visible in the transmitted DIC CLSM channel (LDs).

In live cells, LightOx78 ^46^ displayed very intense lysosomal fluorescence. In fixed cells, intense punctate fluorescence remained; however, these spots did not appear to be lysosomal but rather LDs, as their fluorescence correlated with structures in the transmitted channel, similar to LightOx58.

### 3.6. FluoRaman imaging

The presence of the acetylene stretch from LightOx compounds in the cell-silent region of the spectra indicates their potential as candidates for Raman and FluoRaman microscopy. We imaged these compounds using a Leica SP8 microscopy system equipped with CARS and SRS capabilities.

Although the peak positions of these compounds were previously characterised in various solvents and solid states, minor variations can arise from different experimental setups and environments. Therefore, we measured the SRS spectra in both the solid state (SI Fig. 5) and fixed cells (SI Fig. 6), along with associated controls (SI Fig. S7 and S8). We detected small shifts in the peaks between the solid state and within cells, with peaks observed at 2210 cm^−1^ for both LightOx58 and LightOx78 at concentrations of 50 μM in cells. LightOx73, however, did not show signal above the noise threshold in the SRS spectrum of the cells. To confirm this finding, we measured the spontaneous Raman signal for LightOx73-stained cells and compared it to LightOx58. LightOx58 exhibited a clear peak at approximately 2210 cm^−1^, whereas LightOx73 showed no signal above noise (SI Fig. 9). Unlike LightOx58 and LightOx78, which have bright punctate regions of fluorescence, and high concentrations, LightOx73's fluorescence profile is more diffuse. This indicates that LightOx73's physicochemical profile is more conducive to diffuse localisation throughout the cytoplasm as opposed to concentration into more discrete subcellular compartments as observed with the other compounds.

During all cellular work, we observed punctate structures outside the cells when imaging in the SRS channel tuned for the acetylene stretch. These structures did not show a fluorescent signal, and, unlike the LightOx compounds, they did not produce sharp Raman peaks; instead, they yielded a much broader signal, which allowed for some suppression through off-resonance subtraction. Given their presence in both live and fixed controls, these structures were likely an artefact of the cell line, potentially representing extracellular vesicles expelled by the cells (SI Fig. 7).

**Fig. 7:**
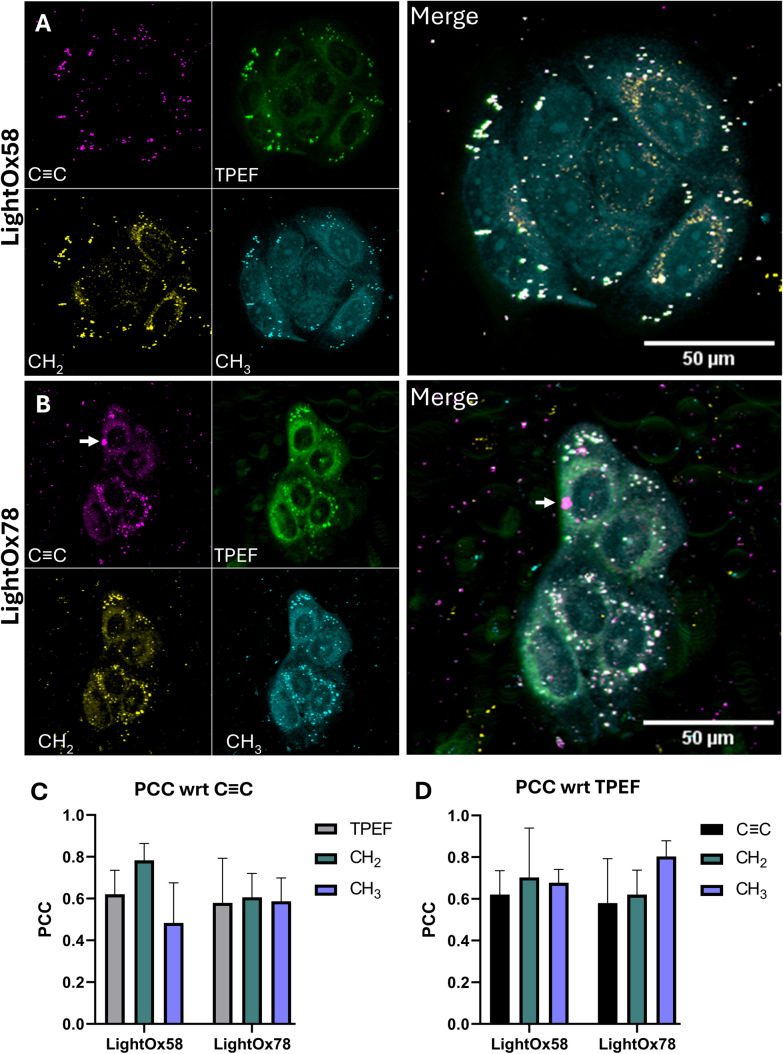
FluoRaman of LightOx compounds. Stimulated Raman scattering (SRS) of C≡C bonds (compound), CH_2_ (cellular lipids), CH_3_ (cellular proteins), and two-photon excitation fluorescence (TPEF) of the compound. 50 μM (A) LightOx58 and (B) LightOx78 in fixed SCC-4 cells. Images are maximum intensity projections from *Z* stacks. (C and D) Pearson's correlation coefficient values between the microscopy channels.

For each LightOx compound, SRS was measured at approximately 2210 cm^−1^ (C≡C), 2850 cm^−1^ (CH_2_ lipids), 2945 cm^−1^ (CH_3_ lipids and proteins),^41^ and 2650 cm^−1^ (off-resonance), alongside two-photon excitation fluorescence (TPEF) (Fig. 7). Subsequently, Pearson's Correlation Coefficient (PCC) analysis was conducted between the microscopy images (Fig. 7C and D).

When exemplifying a technique operating close to its limit of detection, ensuring reproducibility is critical. Biological variability in protein and lipid content, growth conditions, and metabolic state can influence CH_2_ and CH_3_ stretching signals, leading to intercellular and inter-replicate differences that are inherent to all live-cell experiments. To assess reproducibility, three datasets were acquired for each compound, which showed consistent results within the same cell line.

A strong C≡C Raman signal was exhibited in the lipid droplets (LDs) of cells stained with the highly lipophilic LightOx58 (Fig. 7A), which colocalised with punctate spots from the other three channels. Notably, the highest PCC for C≡C was observed with the CH_2_ stretch, rather than the TPEF channel. While it's unsurprising that the C≡C signal strongly localises with the CH_2_ stretch of fatty acids, this finding highlights a discrepancy between the Raman and fluorescence signals of LightOx58. Although the SRS signal is linear with concentration, any signal below the noise threshold will be excluded from the final image once off-resonance subtraction is performed. Therefore, the signal observed in the *peri*-nuclear regions of the TPEF channel might not be present in the C≡C channel because the local concentration was insufficient to yield a detectable Raman signal.

The C≡C Raman signal of LightOx78 (Fig. 7B) displayed bright punctate spots that colocalised across the other three channels. Although these spots were more heterogeneous in size compared to those observed *via* confocal microscopy, the larger spots are likely multiple unresolved LDs. In addition to the intense signal in LDs, *peri*-nuclear Raman signal was also observed in the C≡C channel, indicating that the local concentration of compound in these regions was sufficient to overcome the noise threshold. The arrow in the C≡C channel (Fig. 7B) indicates a large bright feature. As this feature is not observed in the other channels, it is likely a compound aggregate where fluorescence is quenched. Such aggregates can form when the compound concentration significantly exceeds its measured aqueous kinetic solubility.

## 4. Discussion

### 4.1. LightOx compounds as lipophilic FluoRaman probes

Raman microscopy offers a less environment-sensitive method for accurate spatial mapping of compound localisation compared to fluorescence-based methods. This capability holds immense promise for tracking small molecule and drug uptake in a wide array of research and biomedical applications.^30^ Despite this potential, the inherent weakness of the Raman signal makes obtaining results above noise thresholds challenging.

Of the molecules tested, successful imaging was achieved with LightOx58 and LightOx78 using FluoRaman. These experiments required concentrations of 50 μM. This is a typical concentration for small molecules tagged with alkynes, which generally ranges from 10–100 μM,^52–57^ however it is significantly higher than those used in previous work and exceeds the aqueous kinetic solubility of these compounds. While these concentrations present potential toxicity and aggregation issues and limit live-cell applications, our findings clearly demonstrate the feasibility of LightOx58 and LightOx78 as FluoRaman probes for future applications.

The physicochemical properties of LightOx compounds clearly facilitate high local concentrations *in cellulo*. Both LightOx58 and LightOx78 exhibited intense punctate spots in both their Raman and fluorescence signals, showing a high degree of correlation between the two. In contrast, LightOx73 did not produce any detectable Raman signal above the noise threshold. This is likely because LightOx73 is less lipophilic, which reduces its tendency to form highly localised concentrations in lipid-rich subcellular regions, thereby hindering the accumulation necessary for a strong Raman signal.

The observed discrepancy in the cellular localisation of LightOx78 between live (lysosomal) and fixed cells (LDs) may be due to fixation-induced modifications. LightOx78 has a secondary amine, which could potentially react with paraformaldehyde (PFA), either through the formation of imines or *via* Mannich or Eschweiler–Clarke reactions.^58^ Such modifications might alter the compound's lipophilicity, driving the observed changes in localisation and fluorescence. Alternatively, the contrasting fluorescence could arise from fixation-induced changes in the microenvironment of subcellular organelles.

The concentrations required currently limit the feasibility of these molecules for live biological applications using Raman. Toxicity mitigation can be approached from both sample and experimental perspectives. From a sample standpoint, increasing the Raman cross section, such as the incorporation of a diyne,^59^ or improving aqueous solubility to prevent probe precipitation, can reduce the required probe concentration while improving cellular tolerability. Experimentally, enhancing system sensitivity enables the use of lower laser powers and shorter exposure times, with recent advances in coherent Raman scattering, including quantum noise reduction^60^ and electronic resonance enhancement,^61^ further supporting the use of sub-toxic probe concentrations in live-cell experiments.

### 4.2. Environmental sensors

The photophysical characteristics of LightOx compounds are highly dependent on environmental factors such as temperature, pH, viscosity, and polarity. Each subcellular region has its own unique microenvironment, leading to variations in fluorophore localisation, emission, and lifetime throughout the cell.

The FLIM images (Fig. 3) revealed a large diversity in LightOx lifetimes across the cell. The longest lifetimes were consistently found in the plasma membrane, a lipophilic non-polar region.^62^ The aqueous cytosol exhibited the shortest lifetimes, with the ER and Golgi regions displaying intermediate values. Excluding very non-polar environments, this observation is consistent with our *ex cellulo* experimental findings.

LightOx58, a highly lipophilic compound known to localise in LDs, exhibited a comparatively narrow lifetime histogram, with a sharp peak at approximately 2.15 ns corresponding specifically to lipid droplets. While LDs are highly lipophilic, non-polar environments, one might intuitively expect a comparatively longer lifetime. However, LightOx58's lifetimes in LDs are shorter than those observed in the ER/nuclear membrane and plasma membrane regions. Given that intersystem crossing (ISC) most readily occurs in non-polar environments,^46^ it is likely that this process is efficiently occurring within the LDs, thereby reducing the fluorescence lifetime *via* non-radiative decay.

Lysosomes are primarily recognised as degradative organelles, with additional roles in signalling and metabolism.^63^ They are characterised by significant heterogeneity in size (0.1–1.2 μm), pH (4.5–5.5), morphology, and composition.^63,64^ A study by Zhanghao and coworkers.^62^ indicated that lysosomal membranes are less polar than LDs, although with a greater degree of variability. Furthermore, pH is known to vary between juxtanuclear and peripheral lysosomes,^63^ with juxtanuclear lysosomes (involved in endocytic, autophagic, and phagocytic lysosome reformation) generally being larger and more acidic.

Previous pH studies^36^ demonstrated that radiative emission (quantum yield, QY) increases with increasing pH for similar alkyl amine structures. As lifetime generally increases with increasing QY (due to the energy gap law), it might be expected that juxtanuclear lysosomes would exhibit shorter lifetimes compared to their more alkaline peripheral counterparts. Qualitatively, this aligns with our observations of the lifetimes of LightOx78 in lysosomes (Fig. 3D). Shorter lifetimes are measured in lysosomes (red/orange/yellow) predominately closer to the nucleus, while longer lifetimes are measured in lysosomes (blue/green) that tend to be more peripheral. Additionally, other factors such as ISC efficiency will also influence the lifetime; lysosomes that are more amenable to this process will exhibit shorter fluorescence lifetimes.

The compounds lacking distinct punctate localisation, LightOx17 and LightOx73, exhibited broad cellular localisation with contrasting lifetimes. LightOx73, the least lipophilic compound, showed two distinct lifetimes: a sharp peak at approximately 3 ns corresponding to the plasma membrane and a shorter-lived peak associated with cytosolic localisation. Within the cytosolic region, there appeared to be heterogeneity, which may correlate with distinct subcellular regions.

LightOx17 possesses a very high log *P* (partition coefficient, measure of lipophilicity) value, which would typically suggest localisation to non-polar, lipophilic areas, and thus predict longer lifetimes. However, at physiological pH (∼7), the carboxylic acid moiety of LightOx17 will be deprotonated, leading to reduced lipophilicity compared to its neutral, uncharged form (SI Fig. 10). We previously reported that the corresponding methyl ester, which retains high lipophilicity at physiological pH (SI Fig. 10), localises to LDs.^36^ Therefore, deprotonation directs the localisation of LightOx17 to less lipophilic and more polar regions, which may explain the shorter lifetimes observed *in cellulo*.

The environmental sensitivity of fluorescence lifetime in these molecules highlights their potential to reveal key micro-environmental information. When combined with stimulated Raman spectroscopy, this could enable precise spatial mapping with concentration measurements – something not achievable with FLIM alone. Furthermore, the broad emission profiles of this class of molecules suggest their potential utility to investigate drug interactions using FLIM–FRET techniques, offering an additional layer of mechanistic insight.

### 4.3. Subcellular dependency of inter-system crossing

Previous studies have elucidated the environmental dependency of intersystem crossing (ISC) within this group of molecules, demonstrating that triplet-formation is most efficient in non-polar environments. Phosphorescence imaging thus holds potential as a tool that could provide a means to examine ISC efficiency across distinct subcellular regions with a well-calibrated probe. In both normoxic and hypoxic conditions, the majority of the detectable phosphorescence signal occurred in the *peri*-nuclear regions (likely encompassing the endoplasmic reticulum, Golgi apparatus, and lysosomes), correlating with the fluorescence signal, presumably due to the relatively higher concentration of compound in these subcellular loci.

In the cellular environment, factors such as temperature, polarity, and relative concentrations of quenching moieties can significantly influence triplet decay and phosphorescence lifetime.^65^ In more lipophilic, non-polar environments, longer phosphorescence lifetimes would generally be expected due to suppressed vibrational relaxation, reduced oxygen availability, and adherence to the energy gap law.^42^

The most intense phosphorescence of LightOx78 under hypoxic conditions was observed in the *peri*-nuclear region (highlighted with an arrow in Fig. 5, and in SI Fig. 3). In this specific region, the fluorescence lifetime was comparatively short, which aligns with increased non-radiative decay to the triplet manifold (*i.e.*, enhanced ISC). Concurrently, this same area corresponded to longer phosphorescence lifetimes than those of the surrounding subcellular regions. In the context of photosensitisation, long phosphorescent lifetimes are important because extended excited-state durations are conducive to greater reactive oxygen species (ROS) production and consequently, enhanced therapeutic effects.^66^

As discussed previously (Fig. 3), juxtanuclear lysosomes tended to exhibit shorter fluorescence lifetimes compared to their peripheral counterparts. Although juxtanuclear lysosomes are not inherently more lipophilic, their increased acidity could enhance non-radiative decay mechanisms. Therefore, it appears that these more acidic juxtanuclear lysosomes are particularly conducive to efficient intersystem crossing.

## 5. Conclusions

We have demonstrated the potential of integrating complementary imaging modalities: fluorescence; FLIM; PLIM; and Raman/FluoRaman microscopy, to obtain multifaceted information from cellular systems. By combining environmental sensitivity (*via* FLIM), oxygen-responsive triplet emission (*via* PLIM), and vibrational specificity (*via* Raman/FluoRaman), we exemplify multimodal imaging capable of probing subcellular environments with high spatial and functional resolution.

Using four representative diarylacetylene-based fluorophores as exemplars, we revealed how differences in localisation and photophysical behaviour can be effectively captured across these modalities. FLIM revealed broad localisation and pronounced environmental sensitivity, while PLIM identified regions of elevated intersystem crossing that correlated with shorter fluorescence lifetimes. FluoRaman microscopy further enabled label-specific visualisation of compounds exhibiting distinct punctate localisation.

Raman microscopy proved effective only for compounds that accumulate at highly localised concentrations, representing a limitation for broader applicability. Continued advances in probe development, such as increasing Raman cross-sections and tuning physicochemical properties, alongside improvements in imaging sensitivity, will further enhance this workflow. Overall, the integration of these complementary techniques establishes a foundation for powerful multimodal imaging strategies in biomolecular analysis and drug discovery.

## Author contributions

Conceptualization – DRC, CAA, NAB, MS, SWB; data curation – JGH, CD, DT, AEB; formal analysis – JGH, CD, DT, AEB; funding acquisition – DRC, CAA, SWB, NAB, HHF; investigation – JGH, CD, DT, AEB, ED; methodology – DRC, NAB, MS, SWB; project administration – DRC, CAA, NAB, MS, SWB; supervision – DRC, CAA, JMG; validation – DRC, CAA, SWB, JMG; visualization – JGH, DT, AEB; writing – original draft – JGH; writing – review & editing – all.

## Conflicts of interest

DRC, JGH, ED, and CAA were employed by the company LightOx Limited. CAA own shares of LightOx Limited, the company licensed to pursue commercial applications of the novel chemicals described in this manuscript.

The remaining authors declare that the research was conducted in the absence of any commercial or financial relationships that could be construed as a potential conflict of interest.

## Supplementary Material

AN-151-D5AN00953G-s001

## Data Availability

Data for this article, including: two-photon cross sections; Raman; FLIM; PLIM; CLSM and FluoRaman are available at Zenodo at https://doi.org/10.5281/zenodo.17056821. Supplementary figures and data detailing multimodal imaging experiments, including lifetime *vs.* viscosity measurements, FLIM, PLIM, CLSM, FluoRaman imaging, and log *D* analyses. Supplementary information (SI) is available. See DOI: https://doi.org/10.1039/d5an00953g.
