# Supplementary material for: Advanced multimodal imaging: FLIM, PLIM, and FluoRaman enabled by novel diarylacetylene probes
Source: Analyst. 2025 Dec 1;151(2):623–35. doi: 10.1039/d5an00953g (PMC12706839; doi:10.1039/d5an00953g)
Supplement: AN-151-D5AN00953G-s001 [file AN-151-D5AN00953G-s001.pdf]

This journal is © The Royal Society of Chemistry 2025

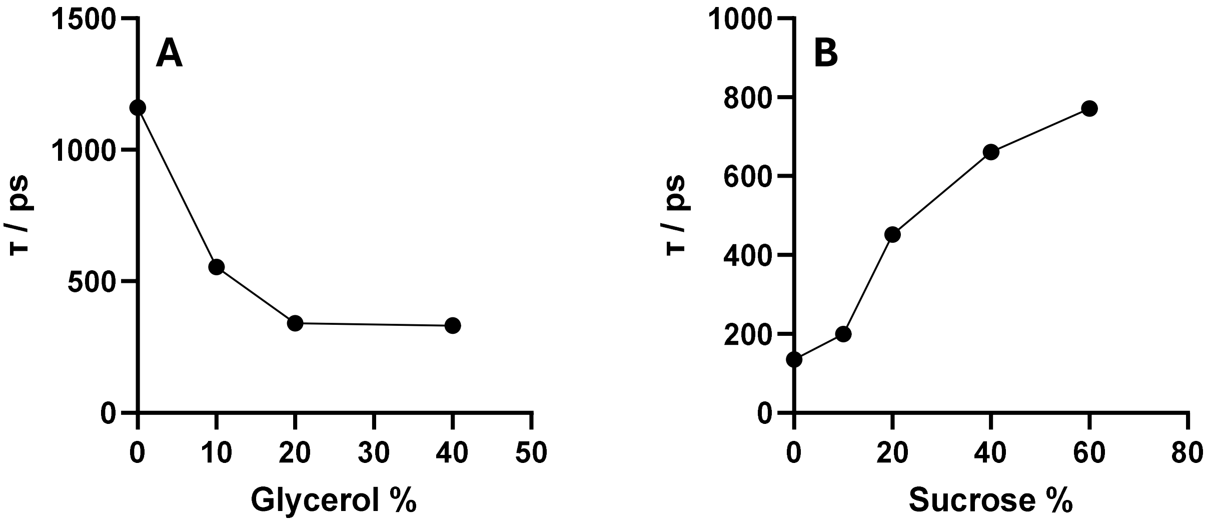


**Fig. 1** Fluorescence lifetime as a function of viscosity. (A) Lifetime of 50 μM LightOx17 in $CHCl_3$ measured using two-photon excitation at 780 nm. (B) Lifetime of 30 μM LightOx17 in water (working concentration made from a stock solution of LightOx17 dissolved in ethanol) measured using one photo excitation at 405 nm.

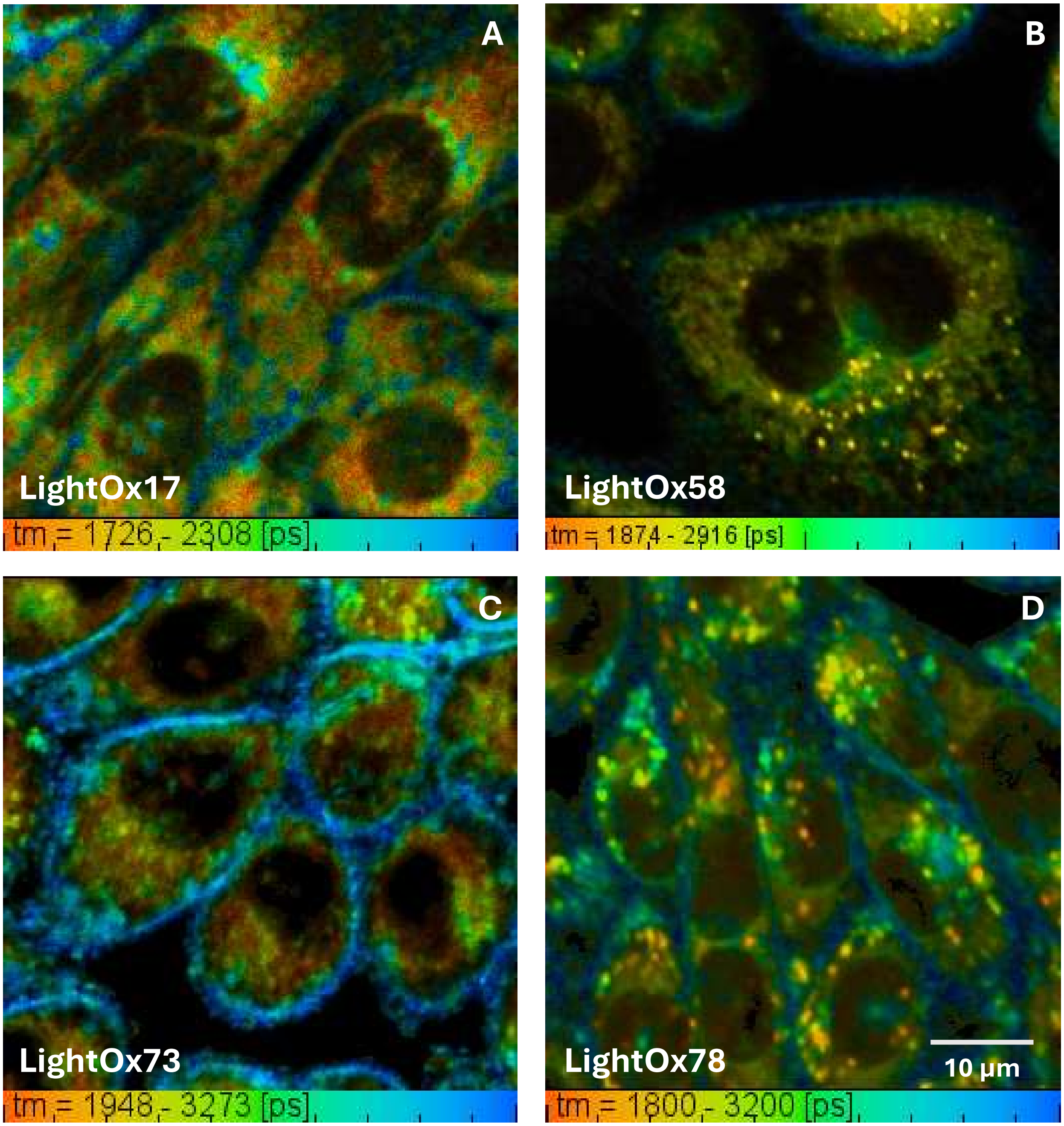


**Fig. 2** Two-photon FLIM of 1 μM (A) LightOx17, (B) LightOx58, (C) LightOx73 and (D) LightOx78 in live CHO cells. Lifetime colour maps were set to the optimal range for each image. The excitation wavelength was 780 nm.

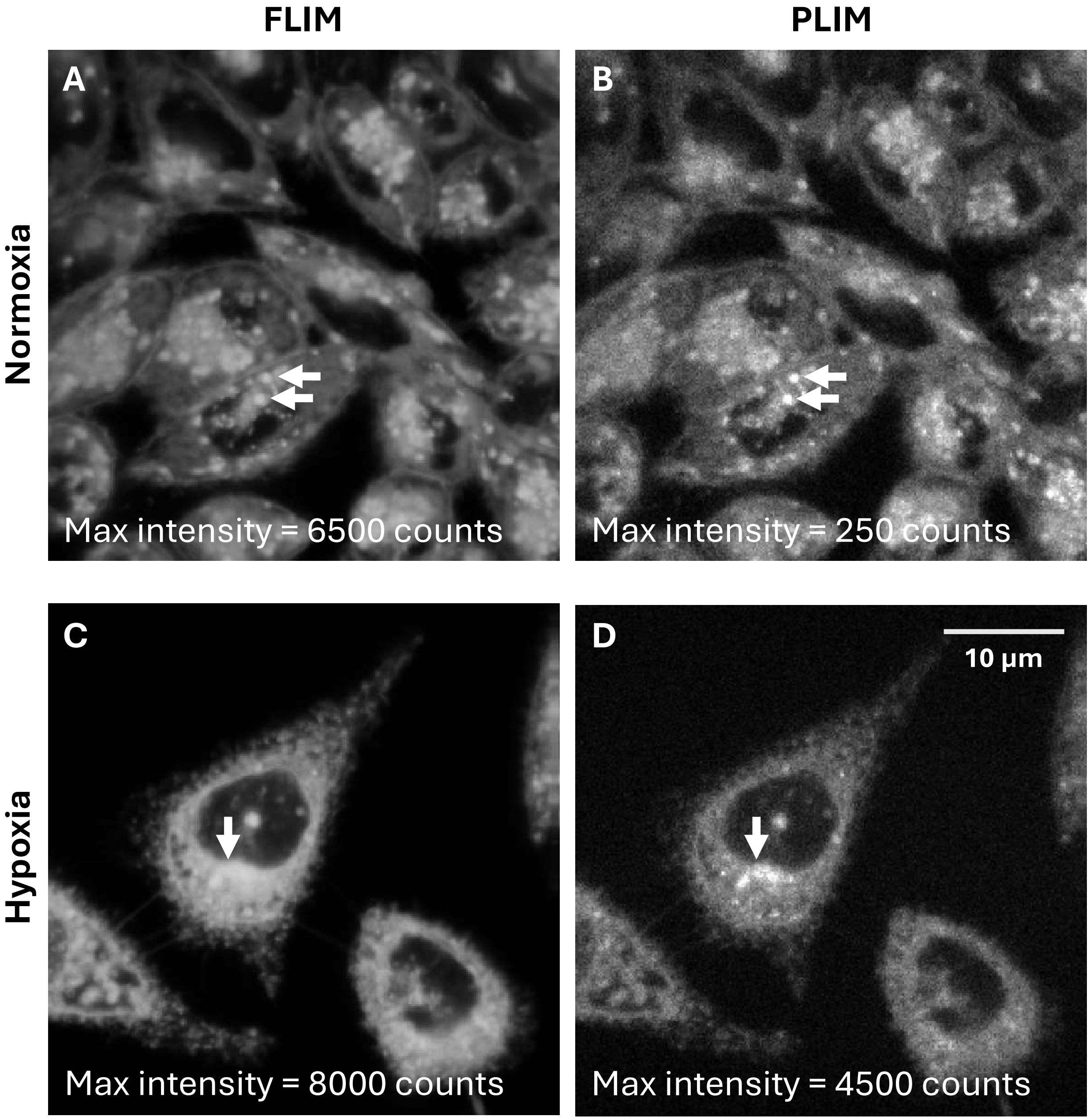


**Fig. 3** Maximum intensity projections of FLIM and PLIM in normoxic and hypoxic conditions. 5 µM LightOx78 in live CHO cells. NB: The intensity projections will have some residual fluorescence counts that cannot be removed.

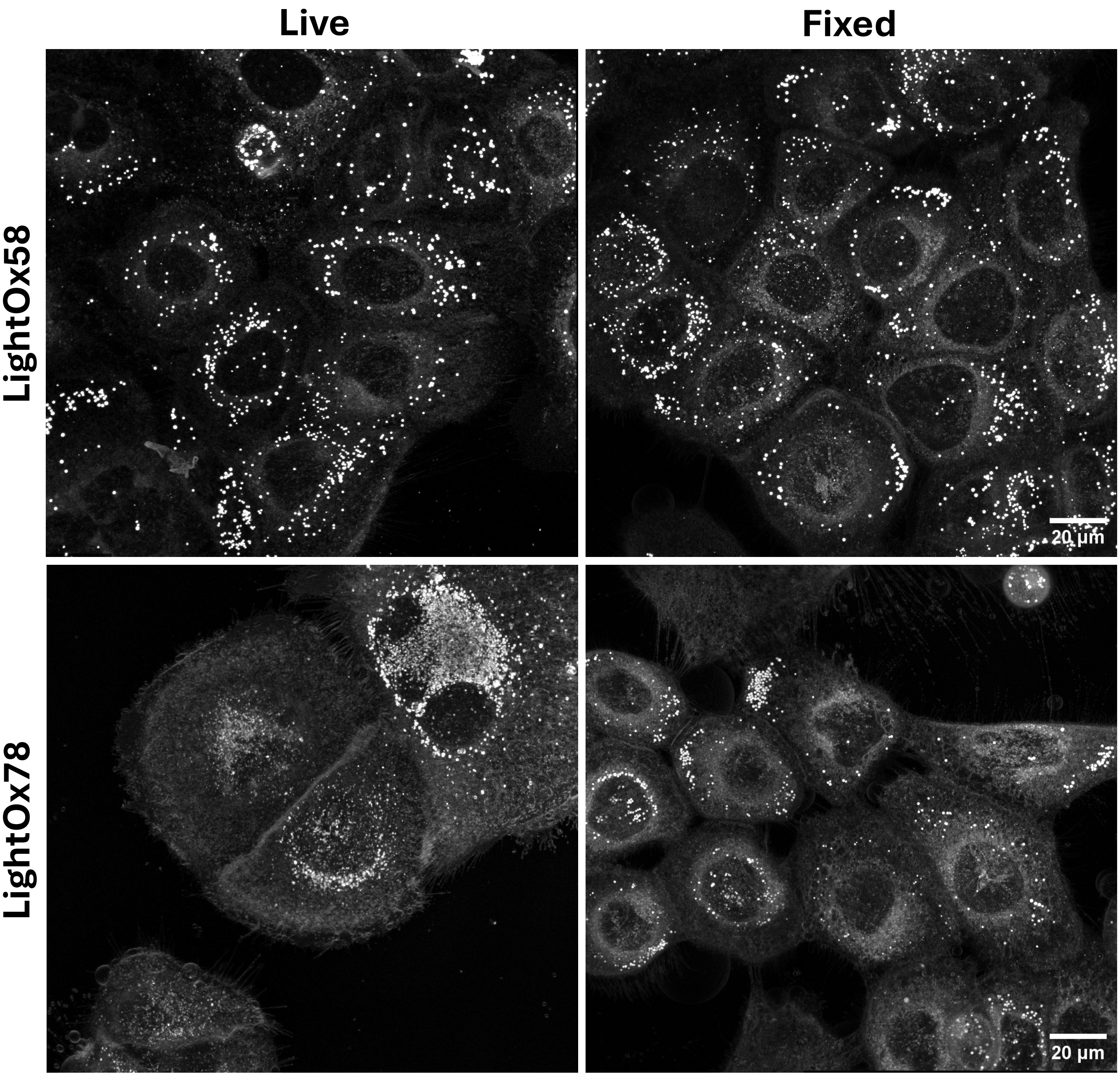


**Fig. 4** CLSM images of 50 µM LightOx58 and LightOx78 in live and fixed (4% PFA) SCC-4 cells.

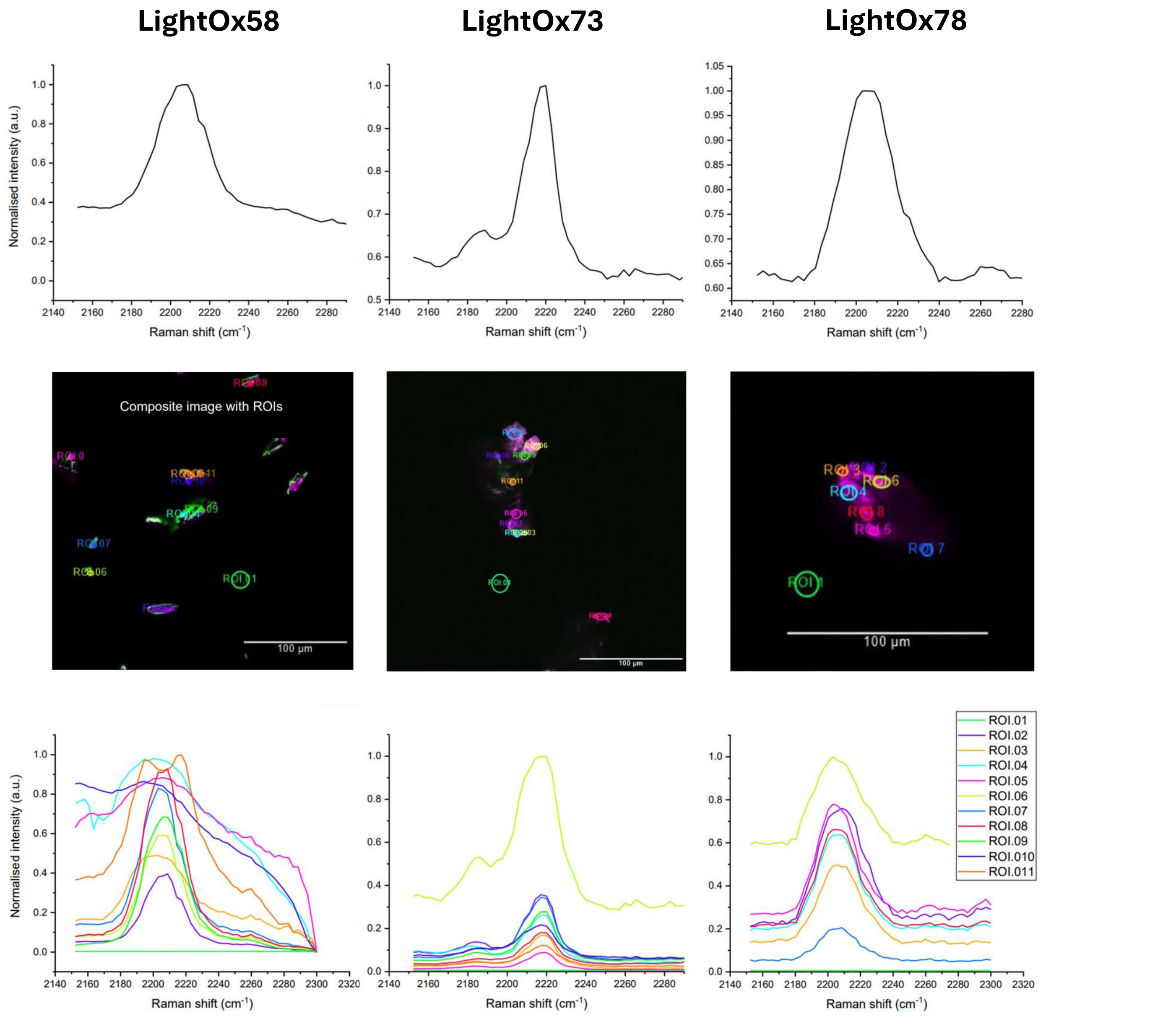


**Fig. 5** SRS spectra of solid state LightOx compounds. The peak centroids were found at 2205, 2218 and 2204 $cm^{-1}$ for LightOx58, LightOx73 and LightOx78 respectively. A smaller peak at 2188 $cm^{-1}$ was also present for LightOx73. Leica SP8 SRS microscope.

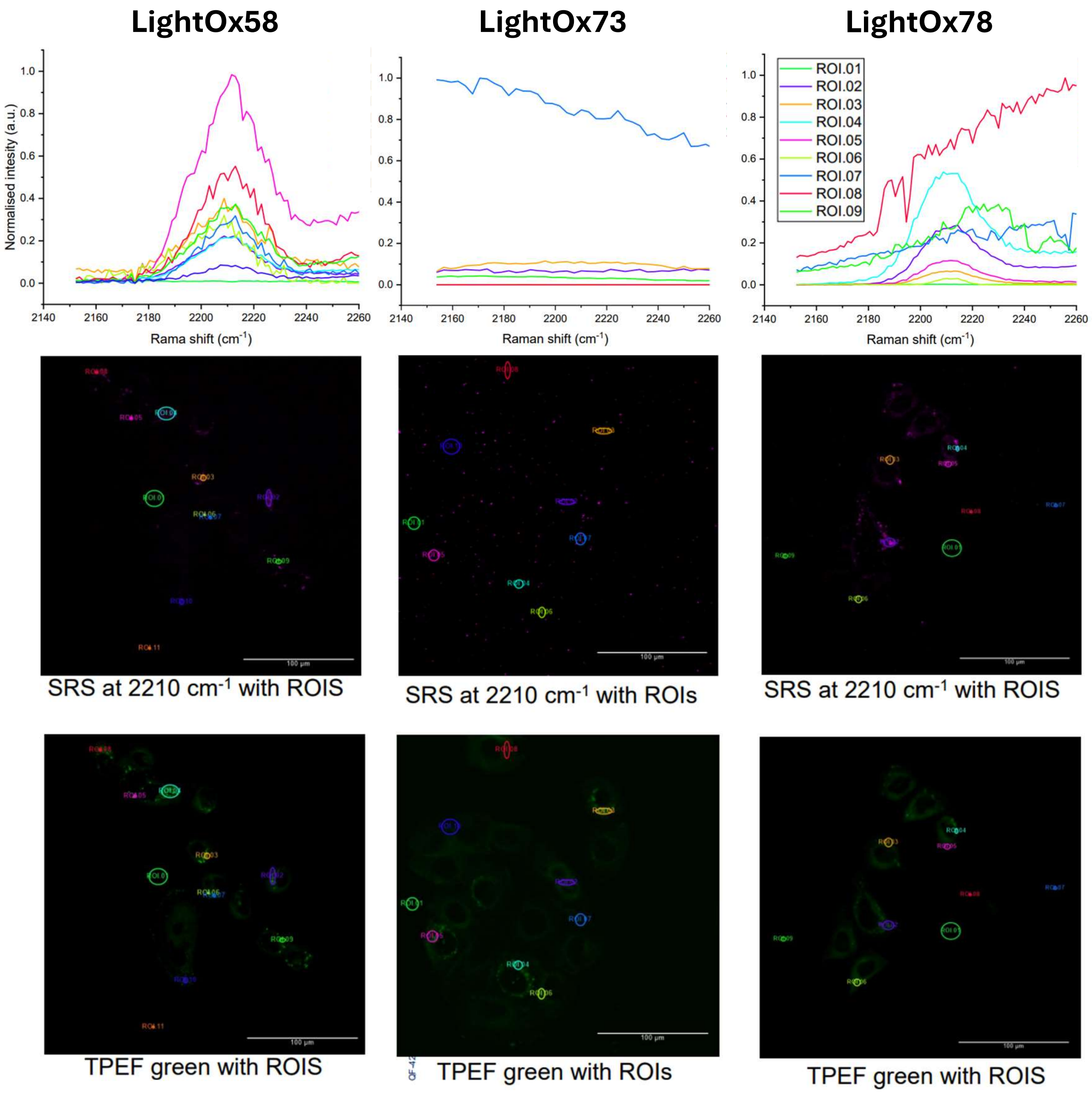


**Fig. 6** SRS spectra of 50 μM LightOx compounds in fixed SCC-4 cells. The peak centroids were found at 2210 cm$^{-1}$ for both LightOx58 and LightOx78. No signal above noise was detected for LightOx73. Leica SP8 SRS microscope

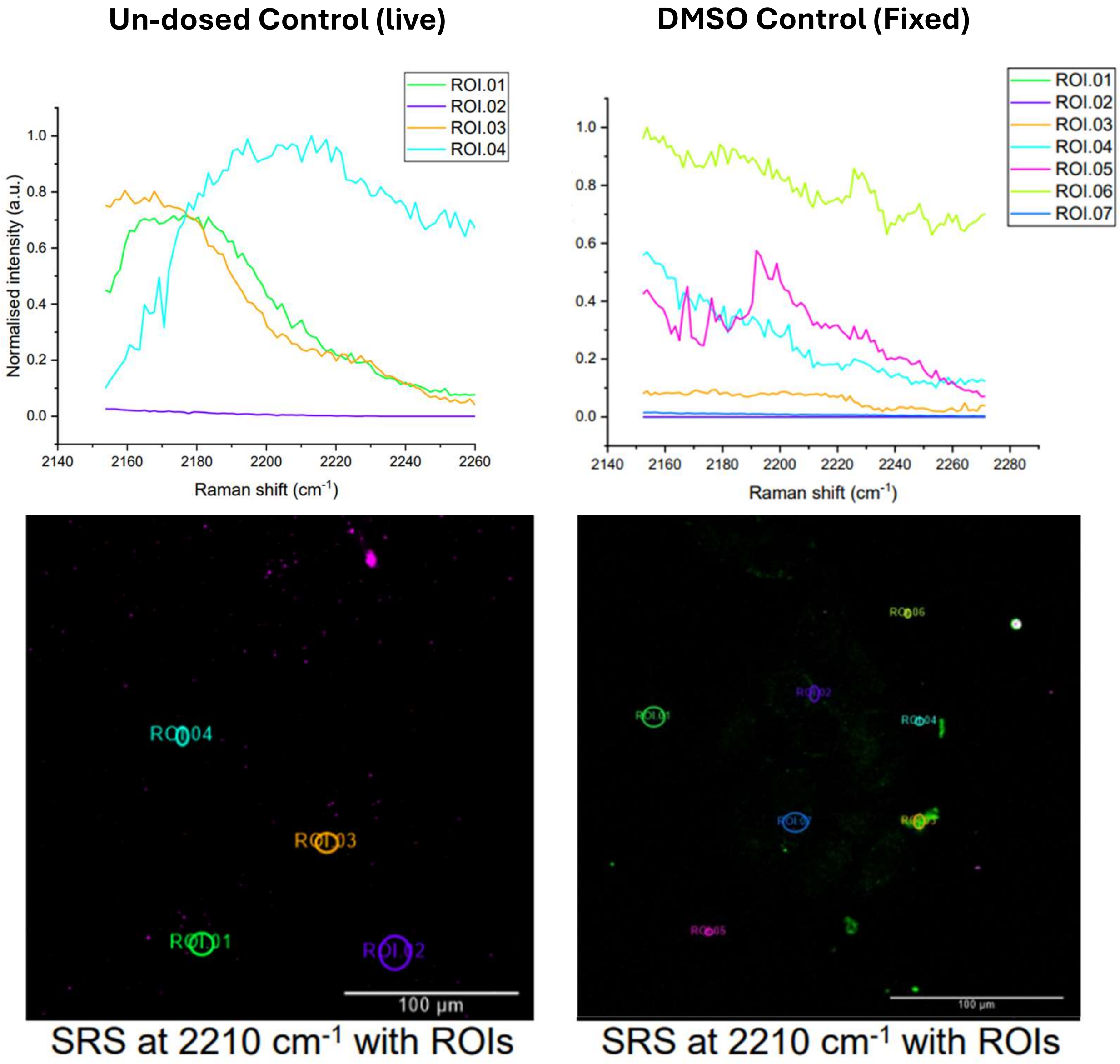


**Fig. 7** SRS spectra of controls (un-dosed live cells and DMSO treated fixed cells). Leica SP8 SRS microscope.

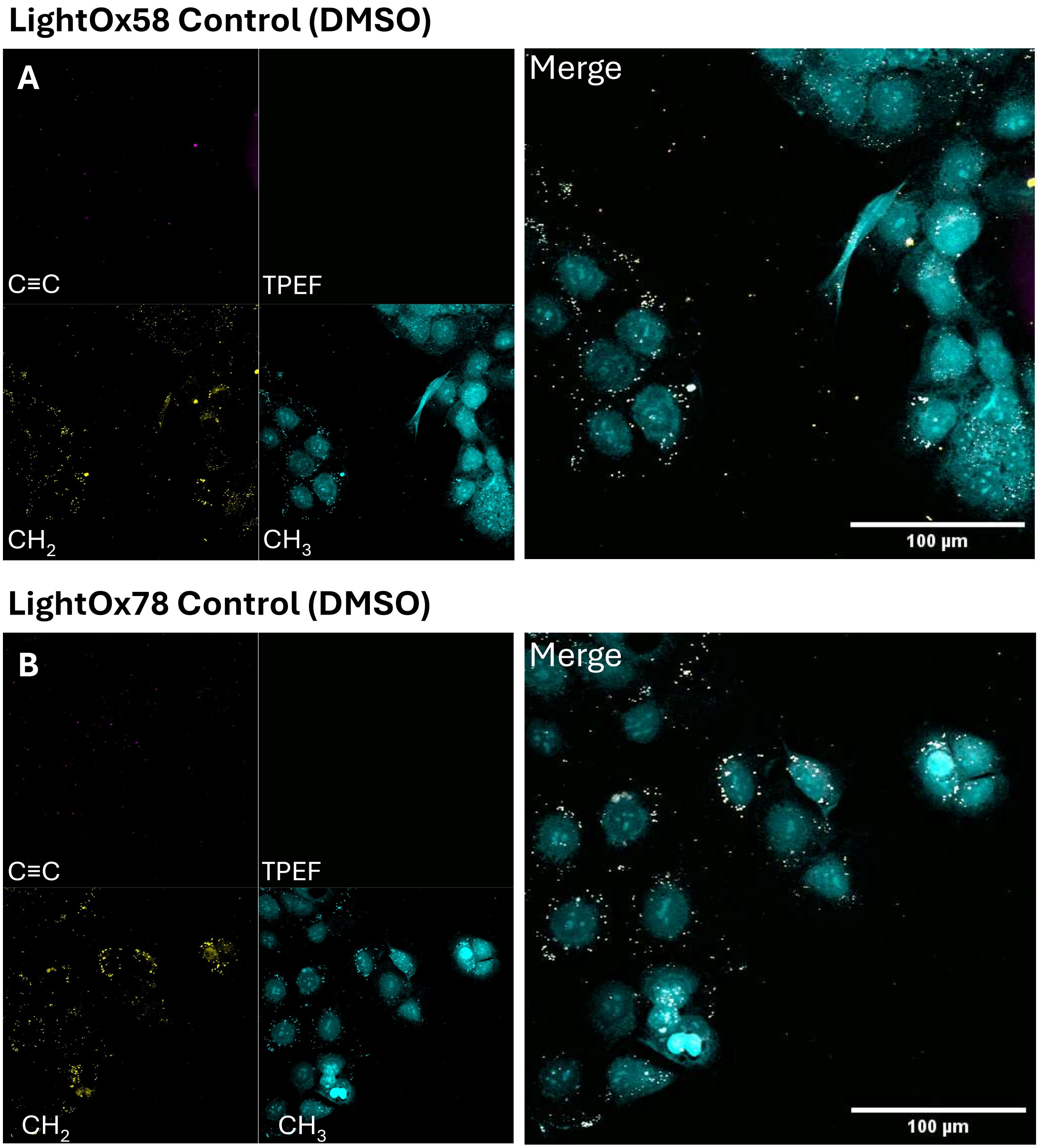


**Fig. 8** DMSO control using the SRS wavenumber used for each LightOx compound. SRS of C≡C bonds (compound), $CH_2$ (cellular lipids), $CH_3$ (cellular proteins), and TPE fluorescence of the compound. 0.5% DMSO used instead of LightOx compound. The same pump and probe laser wavelengths and powers were used for both the sample and corresponding control: (A) 2210 $cm^{-1}$ for LightOx58 and (B) 2210 $cm^{-1}$ for LightOx78.

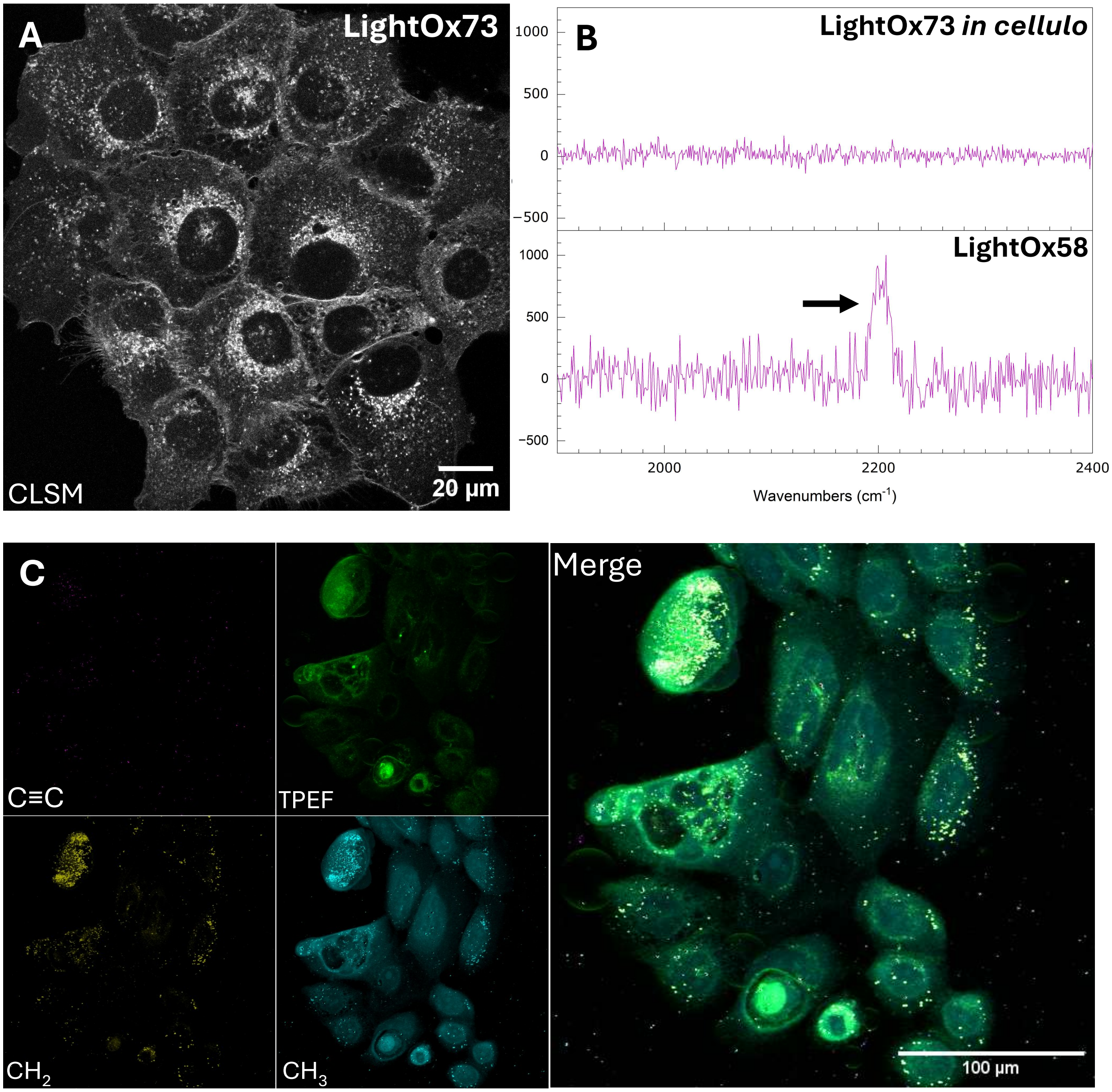


**Fig. 9** FluoRaman of 50 μM LightOx73. (A) CLSM. (B) Continuous wave Raman signal from cells incubated with 50 μ LightOx58 and LightOx73 respectively. (C) SRS of C≡C bonds (2210 $cm^{-1}$, compound), $CH_2$ (2840 $cm^{-1}$, cellular lipids), $CH_3$ (2950 $cm^{-1}$, cellular proteins), and two-photon fluorescence (TPEF) of the compound.

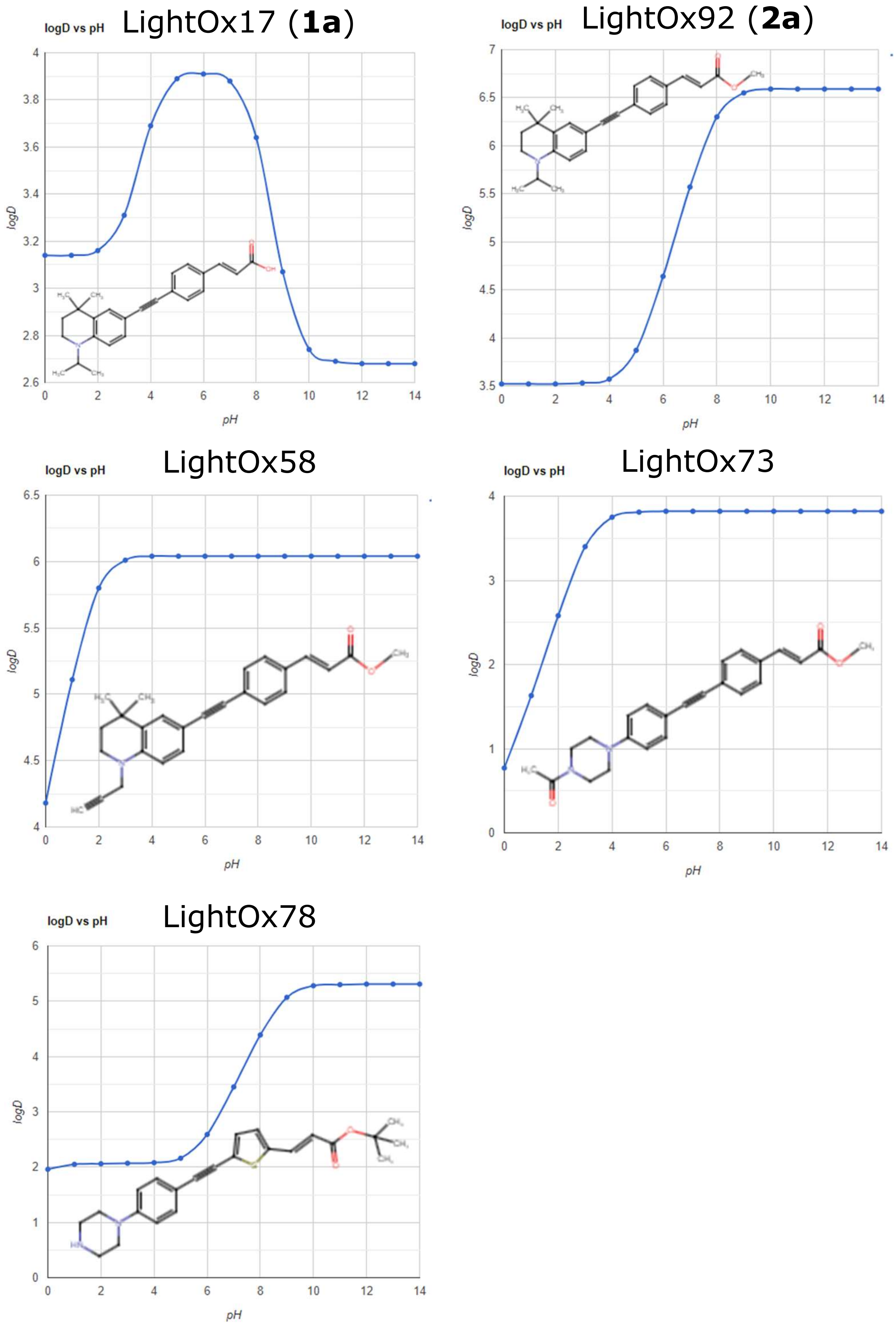


**Fig. 10** Log $D$ (lipophilicity) verse pH for LightOx17, LightOx92, LightOx58, LightOx73, and LightOx78.
